# Association of Thyroid Function with Blood Pressure and Cardiovascular Disease: A Mendelian Randomization

**DOI:** 10.3390/jpm11121306

**Published:** 2021-12-06

**Authors:** Alice Giontella, Luca A. Lotta, John D. Overton, Aris Baras, on behalf of Regeneron Genetics Center, Andrea Sartorio, Pietro Minuz, Dipender Gill, Olle Melander, Cristiano Fava

**Affiliations:** 1 Department of Medicine, University of Verona, 37134 Verona, Italy; alice.giontella@univr.it (A.G.); andrea.sartorio@studenti.univr.it (A.S.); pietro.minuz@univ.it (P.M.); 2 Clinical Research Center, Department of Clinical Sciences, Lund University, 214 28 Malmö, Sweden; olle.melander@med.lu.se; 3 Regeneron Genetics Center, Tarrytown, NY 10591, USA; luca.lotta@regeneron.com (L.A.L.); john.overton@regeneron.com (J.D.O.); aris.baras@regeneron.com (A.B.); 4 Department of Epidemiology and Biostatistics, Imperial College London, London SW7 2AZ, UK; dipender.gill@imperial.ac.uk; 5 Novo Nordisk Research Centre Oxford, Old Road Campus, Oxford OX3 7FZ, UK; 6 Clinical Pharmacology Group, Pharmacy and Medicines Directorate, St. George’s University Hospitals NHS Foundation Trust, London SW17 0QT, UK; 7 Clinical Pharmacology and Therapeutics Section, Institute for Infection and Immunity, St George’s, University of London, London SW17 0RE, UK; 8 Department of Emergency and Internal Medicine, Skåne University Hospital, 214 28 Malmö, Sweden

**Keywords:** thyroid, genetics, polymorphisms, Mendelian randomization, hypertension, cardiovascular diseases

## Abstract

Thyroid function has a widespread effect on the cardiometabolic system. However, the causal association between either subclinical hyper- or hypothyroidism and the thyroid hormones with blood pressure (BP) and cardiovascular diseases (CVD) is not clear. We aim to investigate this in a two-sample Mendelian randomization (MR) study. Single nucleotide polymorphisms (SNPs) associated with thyroid-stimulating hormone (TSH), free tetraiodothyronine (FT4), hyper- and hypothyroidism, and anti-thyroid peroxidase antibodies (TPOAb), from genome-wide association studies (GWAS), were selected as MR instrumental variables. SNPs–outcome (BP, CVD) associations were evaluated in a large-scale cohort, the Malmö Diet and Cancer Study (n = 29,298). Causal estimates were computed by inverse-variance weighted (IVW), weighted median, and MR-Egger approaches. Genetically increased levels of TSH were associated with decreased systolic BP and with a lower risk of atrial fibrillation. Hyperthyroidism and TPOAb were associated with a lower risk of atrial fibrillation. Our data support a causal association between genetically decreased levels of TSH and both atrial fibrillation and systolic BP. The lack of significance after Bonferroni correction and the sensitivity analyses suggesting pleiotropy, should prompt us to be cautious in their interpretation. Nevertheless, these findings offer mechanistic insight into the etiology of CVD. Further work into the genes involved in thyroid functions and their relation to cardiovascular outcomes may highlight pathways for targeted intervention.

## 1. Introduction

The influence of thyroid hormones on the cardiovascular system is well-established [1]. Thyroid hormones exert direct or indirect effects on the sympathetic nervous system and peripheral circulation [2], on cardiomyocytes, and on vascular smooth cells [3]. Abnormalities in their concentrations have profound effects on the cardiometabolic and hemodynamic systems [3]. When primary hypothyroidism occurs, the thyroid-stimulating hormone (TSH) concentration is usually higher than 10 mU/L, while primary hyperthyroidism is characterized by a low TSH level (usually less than 0.01 mU/L). Both conditions can be defined as clinical or subclinical if they are, respectively, combined or not with altered free tetraiodothyronine (FT4) [4]. Indeed, both subclinical hyperthyroidism and hypothyroidism have been reported to be associated with increased systolic blood pressure (SBP) and diastolic blood pressure (DBP) [1,5,6], and with increased cardiovascular risk. In particular, the link between hyperthyroidism (even subclinical) and the risk of atrial fibrillation has been widely reported [7,8,9]. Anti-thyroid peroxidase antibodies (TPO-Ab) are auto-antibodies responsible for autoimmune thyroid disease and have been associated with subclinical atherosclerosis [10,11]. At the genomic level, thyroid hormone target genes are involved in different cellular pathways, such as gluconeogenesis, insulin signaling, and lipogenesis [12]. The complex regulation system, of which the thyroid is part, makes it difficult to determine targeted associations with specific traits and this is also reflected in the different panel of genes and single nucleotide polymorphisms (SNPs) that have been identified by genome-wide association studies (GWASs) [13]. We can take advantage of such genetic information to infer causality between an exposure trait and outcomes using the Mendelian randomization (MR) technique, which exploits the property of alleles to be randomly distributed among the population, allowing an estimation that overcomes the effect of cofounding factors [14]. Hence, in this study, we used a two-sample MR approach to evaluate whether a causal association exists between TSH, FT4, hyperthyroidism, hypothyroidism, and TPOAb with blood pressure traits and cardiovascular events in a Swedish cohort, the Malmö Diet and Cancer Study. We selected only those variants that have already been reported to be associated with the traits in the latest GWAS studies [15,16]. To date, there are no clear data about the causal association between thyroid function and blood pressure traits or adverse cardiovascular events, so we aim to provide further knowledge in this field.

## 2. Materials and Methods

### 2.1. Mendelian Randomization

A two-sample MR study uses summary-level data estimates from SNPs–exposure and SNPs–outcome associations computed in two independent populations [17]. The causal estimates are then assessed by the inverse variance weighted (IVW) method in which the association between the genetic instruments and the outcome is divided by the association of the genetic instruments with the exposure [17]. The analysis was performed using the MR-STROBE guidelines as the reference for reporting MR studies [18].

### 2.2. Association with Exposures

Single nucleotide polymorphisms (SNPs) and summary statistics associated with TSH, FT4, hypothyroidism, hyperthyroidism, and TPOAb were selected from the latest GWAS studies [16,19] to be used as instrumental variables for the two-sample MR analysis. In Supplementary Materials, Table S1, more details about the GWAS are summarized.

Only uncorrelated SNPs (r^2^ < 0.01) that were associated with the traits at the GWAS significance level (*p* < 5 × 10^−8^) were included in the analysis. SNPs with palindromic alleles and with intermediate allele frequencies were excluded.

We selected 52 SNPs for TSH, 29 for FT4, 8 for hypothyroidism and hyperthyroidism and 4 for TPOAb as instrumental variables for the analysis. A list of the variants is shown in Supplementary Materials, Table S2.

### 2.3. Association with the Outcomes

The association of the genetic instruments with the outcomes was evaluated using genotypes from a large-scale Swedish urban-population-based cohort study: the Malmö Diet and Cancer (MDC) Study [20]. The MDC involved 30,447 individuals (58 ± 7.6 years) from Malmö (Sweden) between 1991 and 1996 [20] of whom 29,386 were genotyped for GWAS [21]. A description of the population is included in Supplementary Materials, Table S3.

### 2.4. Outcome Phenotypes

Blood pressure was measured once in the supine position after a 5 min rest. Blood pressure values were adjusted for the number of antihypertensive (AHT) drugs, using a step increase of 8/4, 14/10, 20/16, and 26/22 mmHg SBP/DBP in the presence of 1, 2, 3 or 4 medications, respectively, as previously described [22]. Cardiovascular endpoints referred to a follow-up time of 19.7 ± 5.6 years and consisted of the following categories based on ICD-9 and ICD-10 classifications: cardiovascular disease (the sum of coronary artery diseases and stroke), stroke, coronary artery disease (the presence of coronary events, coronary artery bypass grafts or percutaneous coronary intervention), heart failure, and atrial fibrillation.

### 2.5. Statistical Analysis

Linear and logistic regression models were used to compute the association of the genetic variants with continuous traits (age, sex. and AHT-adjusted systolic and diastolic blood pressure) and with dichotomized events (cardiovascular diseases), respectively. Potential causality was evaluated using the inverse variance weighted (IVW) method, and the weighted median and MR-Egger methods were used in sensitivity analyses to explore bias introduced by the presence of pleiotropy. Cochran’s Q test was used to infer the presence of heterogeneity, with *p*_het_ < 0.05 suggestive of the presence of heterogeneity among the MR estimates generated by the individual instrumental variables. The presence of outliers was tested using MR-PRESSO [23] and leave-one-out analysis was used to verify if the causal effect would rely on a single variant [24]. The significance was considered *p* < 0.05 or *p* < 0.0007 after Bonferroni multiple testing correction. Analyses were performed by the R software (R Core Team 2019, version 1.3.959, package “TwoSampleMR”) [25,26] and PLINK v. 1.9 [27] (www.cog-genomics.org/plink/1.9/, accessed on 5 October 2021).

## 3. Results

### Association of TSH Levels with Outcomes

A genetically increased level of TSH was found to be significantly associated with a decrease in systolic blood pressure (IVW, beta: −0.83; SE: 0.38; *p*: 0.03) in inverse variance weighted analysis. No other exposure showed an association with blood pressure (Table 1, Supplementary Materials, Figure S1). The causal association was supported in the leave-one-out analysis (Supplementary Materials, Figure S2) but it did not result in being significant at the Bonferroni threshold.

In the evaluation of the association with cardiovascular events, genetically predicted TSH was inversely associated with atrial fibrillation (IVW, beta: −0.204; SE: 0.07; *p*: 0.004). The association was consistent across all three estimators, IVW, MR-Egger (beta: −0.455; SE: 0.169; *p*: 0.009), and weighted median (beta: −0.295; SE: 0.087; *p*: 0.001), as shown in Table 2 and Supplementary Materials Figure S3, and was also maintained in the leave-one-out sensitivity analysis (Supplementary Materials, Figure S4), even if a moderate instrumental heterogeneity was found (IVW, Q = 78.7, df = 51, *p* = 0.008, Supplementary Materials, Table S4). Genetically predicted TPOAb showed an association with atrial fibrillation too, but only using the IVW method (IWV, Beta: −1.48; SE: 0.67; *p*: 0.03). MR-PRESSO did not identify any outliers in the identified analysis. From the evaluation of the MR-Egger intercept, shown in Supplementary Materials Table S5, there was no clear evidence of pleiotropy, as suggested by the *p*-values greater than 0.05.

## 4. Discussion

In this two-sample summary-level MR study, we evaluated the causal association of thyroid function with blood pressure traits and cardiovascular diseases. Single nucleotide polymorphisms associated with TSH, FT4, hyperthyroidism, hypothyroidism, and TPOAb identified from the latest GWAS studies [16,19] were used as instrumental variables. Genetically predicted TSH showed an inverse causal relationship with systolic blood pressure. The link between a decreased level of TSH, of which a decrease to under the normal range is usually associated with hyperthyroidism, and high systolic blood pressure has been largely reported in clinical studies that have found systolic, but not diastolic, blood pressure to be higher in patients with hyperthyroidism [12]. Moreover, the beneficial effects of pharmacological or surgical treatment of hyperthyroidism on systolic blood pressure, up to a decrease of 5 mmHg, have been reported after the normalization of the thyroid hormones [28,29]. In a two-sample MR study, using the same instrumental variables as in our study, an association with genetically approximated TSH levels and pulse pressure was identified, as well as with systolic blood pressure, but after the exclusion of a set of genetic variants [30]. Despite the consistency of the results with the literature, the association remained significant only in IVW analysis, and the significance level did not pass a Bonferroni multiple testing correction. A nonsignificant MR-Egger causal estimate and an intercept different from zero, even if not significant, (beta: −0.05; *p*: 0.39) can be suggestive of pleiotropy [31]. Genetically predicted hyperthyroidism, despite not reaching statistical significance, showed a significant association with SBP for an SNP in gene CAPZB (chromosome 1 p36.13, rs12138950) that is in high linkage disequilibrium (LD, r^2^ > 0.9) with an SNP among those used in the TSH instrumental variables (rs10917469). CAPZB, mapped on chromosome 1, encodes for capping actin protein of muscle Z-line subunit beta, and is related to the response to elevated platelet cytosolic Ca^2+^ and MAPK-ERK pathways (GeneCards identifier: GC01M019339). Among the genetic instruments-cardiovascular outcomes results, we found the associations between TSH and TPOAb through the presence of atrial fibrillation to be statistically significant and clinically relevant. The link between atrial fibrillation and thyroid function has been largely described; moreover, it was reported that atrial fibrillation is reverted in 60% to 75% of patients after they receive antithyroid treatment [32]. The genetically predicted level of TSH was strongly inversely linked to atrial fibrillation and this was confirmed by all the three Mendelian randomization estimators (IVW, MR-Egger and weighted median). Several Mendelian randomization studies, involving large-scale genetic consortia [13,33,34] and longitudinal studies [7,32,35], reported the associations between TSH or hyperthyroidism and atrial fibrillation in line with our results. A low TSH level could affect atrial fibrillation through an increased left ventricular mass and diastolic dysfunction [33,36]. Genetically approximated TPOAb levels were linked to a decreased risk of atrial fibrillation. Among the four SNPs included in the analysis, the one that showed a clearer association with the outcome, rs2010099, is located in the proximity of the gene KALRN (chromosome 3q21.2) that encodes for Kalirin, a guanine nucleotide exchange factor (GEF) in the Rho guanosine triphosphatase (GTPase) signaling pathway, and is involved in smooth muscle cell signaling and migration [37]. It was found to be highly expressed in the left atrium, mitral valve, right atrium and ventricle, and the tricuspid valve and aorta (ENSG00000160145) [37]. This gene was found to be associated with megakaryopoiesis and platelet formation and has also been identified as a target gene for coronary artery disease, diabetes mellitus, coronary calcification, and neointimal hyperplasia [19,37,38,39].

### Strengths and Limitations

A strength of this work is the use of the MR method to infer the causal role of thyroid function on the cardiovascular outcome, in which the effect of cofounding factors is minimized. Moreover, the use of a two-sample MR, in which genetic instrument–exposure and genetic instrument–outcome associations are evaluated in two independent cohorts, minimize the bias introduced by weak instruments [40]. Secondly, a genetic instrument-exposure association from large-scale genomic consortia was used to overcome sample power limitations. Then, the use of genetic variants associated with specific thyroid hormones could help shed light on the way in which each affects specific outcomes.

Among the limitations of this work is the possible presence of horizontal pleiotropy, as suggested from the sensitivity analysis using MR-Egger regression. This could be evidence of the existence of cofounders interfering in the causality evaluation, possibly due to the association of the SNPs with the outcomes through parallel pathways [40]. Further analyses, including multivariable Mendelian randomization, would be needed to clarify which other phenotypes interact in the associations. These could be clarified if even other factors, such as height, can be implied in the causal association [41].

In conclusion, in the present study, we confirm the well-known causal association between genetically decreased TSH and atrial fibrillation. We also report for the first time the association between genetically decreased TSH and systolic blood pressure, which, despite being reasonable, would need further confirmatory results to be definitely clarified. This work offers mechanistic insight and, in the future, a deeper understanding of the specific genes involved in these causal links could allow the development of strategies for targeted intervention.

## Figures and Tables

**Table 1:** Association of THS levels with blood pressure traits.

	IVW			MR Egger			Weighted Median		
TSH									
	β	SE	*p*	β	SE	*p*	β	SE	*p*
SBP	−0.83	0.38	0.03	−1.37	0.92	0.14	−0.74	0.62	0.23
DBP	−0.08	0.51	0.88	−0.12	0.30	0.69	−0.07	0.21	0.74
FT4									
	β	SE	*p*	β	SE	*p*	β	SE	*p*
SBP	0.18	0.31	0.56	−0.22	0.68	0.75	0.12	0.41	0.77
DBP	0.29	0.30	0.33	−0.42	0.66	0.53	0.19	0.40	0.63
Hypothyroidism									
	β	SE	*p*	β	SE	*p*	β	SE	*p*
SBP	−0.56	0.49	0.25	−1.91	2.12	0.40	−0.41	0.47	0.38
DBP	0.11	0.23	0.62	0.16	1.02	0.88	−0.04	0.24	0.86
Hyperthyroidism									
	β	SE	*p*	β	SE	*p*	β	SE	*p*
SBP	0.49	0.25	0.05	0.83	1.27	0.54	0.33	0.033	0.32
DBP	0.10	0.13	0.50	−0.39	0.68	0.59	0.03	0.17	0.84
TPOAb									
	β	SE	*p*	β	SE	*p*	β	SE	*p*
SBP	3.25	5.9	0.59	−38.6	33.2	0.37	−0.64	6.2	0.37
DBP	1.29	3.84	0.74	−34.4	14.6	0.14	−1.9	3.2	0.56

Β, beta coefficient; DBP, diastolic blood pressure; MR, Mendelian randomization; *p*, *p*-value; SE, standard error; SBP, systolic blood pressure; TPOAb, thyroid peroxidase antibodies. Beta coefficient refers to a 1 SD increase in TSH levels. The significance threshold was set to *p* < 0.05 and *p* < 0.0007 after Bonferroni correction.

**Table 2:** Causal association between TSH and cardiovascular diseases.

	IVW			MR Egger			Weighted Median		
TSH									
	β	SE	*p*	β	SE	*p*	β	SE	*p*
CVD	0.03	0.06	0.56	0.11	0.16	0.48	−0.10	0.08	0.89
Atrial fibrillation	−0.204	0.07	0.004	−0.455	0.169	0.009	−0.295	0.087	0.001
Stroke	0.06	0.07	0.40	0.03	0.19	0.89	−0.01	0.10	0.92
FT4									
	β	SE	*p*	β	SE	*p*	β	SE	*p*
CVD	−0.01	0.08	0.87	−0.002	0.18	0.99	−0.02	0.10	0.81
Atrial fibrillation	−0.06	0.09	0.53	−0.17	0.20	0.40	−0.19	0.12	0.10
Stroke	−0.14	0.09	0.11	−0.20	0.20	0.31	−0.23	0.13	0.08
Hypothyroidism									
	β	SE	*p*	β	SE	*p*	β	SE	*p*
CVD	0.02	0.05	0.71	0.03	0.209	0.89	−0.001	0.005	0.98
Atrial fibrillation	−0.56	0.49	0.25	−1.91	2.12	0.4	−0.4	0.48	0.39
Stroke	−0.04	0.07	0.54	−0.01	0.32	0.97	−0.07	0.08	0.37
Hyperthyroidism									
	β	SE	*p*	β	SE	*p*	β	SE	*p*
CVD	−0.01	0.05	0.75	0.06	0.26	0.81	0.007	0.05	0.88
Atrial fibrillation	0.11	0.06	0.08	0.44	0.30	0.19	0.11	0.05	0.03
Stroke	−0.04	0.05	0.45	−0.27	0.297	0.40	−0.02	0.07	0.72
TPOAb									
	β	SE	*p*	β	SE	*p*	β	SE	*p*
CVD	0.40	0.93	0.66	−7.95	3.79	0.17	−0.07	0.82	0.94
Atrial fibrillation	−1.48	0.67	0.03	−6.75	4.09	0.24	−1.16	0.88	0.19
Stroke	−0.06	0.79	0.94	0.72	4.86	0.89	−0.14	0.94	0.88

Β, beta coefficient; CVD, cardiovascular disease; MR, Mendelian randomization; *p*, *p*-value; SE, standard error; TPOAb, thyroid peroxidase antibodies. Beta coefficient refers to a 1 SD increase in TSH levels. The significance threshold was set to *p* < 0.05 and *p* < 0.0007 after Bonferroni correction.

## Data Availability

Data available on request due to restrictions. The data presented in this study are available on request from the corresponding author.

## Supplementary Materials

The following are available online at https://www.mdpi.com/article/10.3390/jpm11121306/s1, Regeneron Genetics Center Banner Author List and Contribution Statements, Figure S1: Causal estimation of TSH on systolic blood pressure. (a) Plot of causal estimate. (b) Funnel plot showing estimates for single variants; Figure S2: Causal estimation of TSH on atrial fibrillation. (a) Plot of causal estimate. (b) Funnel plot showing estimates for single variants; Figure S3: Leave-one-out sensitivity analysis of TSH on SBP; Figure S4: Leave-one-out sensitivity analysis of TSH on AF; Table S1: Characteristics of GWAS from which we selected instrumental variables; Table S2: List of genetic instruments associated to TSH, FT4, Hyperthyroidism, Hypothyroidism and TPOAb; Table S3: Characteristics of the Malmö Diet and Cancer (MDC) cohort; Table S4: Observed heterogeneity computed for observed significant associations (TSH, AF, TPOAb); Table S5: MR-Egger intercept estimation.
