# Supplementary material for: Association of Thyroid Function with Blood Pressure and Cardiovascular Disease: A Mendelian Randomization
Source: J Pers Med. 2021 Dec 6;11(12):1306. doi: 10.3390/jpm11121306 (PMC8704995; doi:10.3390/jpm11121306)

Supplementary Material

Regeneron Genetics Center Banner Author List and Contribution Statements... 1
Supplementary Figures.. 2

Regeneron Genetics Center Banner Author List and Contribution Statements

All authors/contributors are listed in alphabetical order.

RGC Management and Leadership Team
Goncalo Abecasis, Ph.D., Aris Baras, M.D., Michael Cantor, M.D., Giovanni Coppola, M.D., Andrew Deubler, Aris Economides, Ph.D., Luca A. Lotta, M.D., Ph.D., John D. Overton, Ph.D., Jeffrey G. Reid, Ph.D., Alan Shuldiner, M.D.

Contribution: All authors contributed to securing funding, study design and oversight. All authors reviewed the final version of the manuscript.

Sequencing and Lab Operations
Christina Beechert, Caitlin Forsythe, M.S., Erin D. Fuller, Zhenhua Gu, M.S., Michael Lattari, Alexander Lopez, M.S., John D. Overton, Ph.D., Thomas D. Schleicher, M.S., Maria Sotiropoulos Padilla, M.S., Louis Widom, Sarah E. Wolf, M.S., Manasi Pradhan, M.S., Kia Manoochehri, Ricardo H. Ulloa.

Contribution: C.B., C.F., A.L., and J.D.O. performed and are responsible for sample genotyping. C.B, C.F., E.D.F., M.L., M.S.P., L.W., S.E.W., A.L., and J.D.O. performed and are responsible for exome sequencing. T.D.S., Z.G., A.L., and J.D.O. conceived and are responsible for laboratory automation. M.P., K.M., R.U., and J.D.O are responsible for sample tracking and the library information management system.

Genome Informatics
Xiaodong Bai, Ph.D., Suganthi Balasubramanian, Ph.D., Andrew Blumenfeld, Boris Boutkov, Ph.D., Gisu Eom, Lukas Habegger, Ph.D., Alicia Hawes, B.S., Shareef Khalid, Olga Krasheninina, M.S., Rouel Lanche, Adam J. Mansfield, B.A., Evan K. Maxwell, Ph.D., Mrunali Nafde, Sean O'Keeffe, M.S., Max Orelus, Razvan Panea, Ph.D., Tommy Polanco, B.A., Ayesha Rasool, M.S., Jeffrey G. Reid, Ph.D., William Salerno, Ph.D., Jeffrey C. Staples, Ph.D.

Contribution: X.B., A.H., O.K., A.M., S.O., R.P., T.P., A.R., W.S. and J.G.R. performed and are responsible for the compute logistics, analysis and infrastructure needed to produce exome and genotype data. G.E., M.O., M.N. and J.G.R. provided compute infrastructure development and operational support. S.B., S.K., and J.G.R. provide variant and gene annotations and their functional interpretation of variants. E.M., J.S., R.L., B.B., A.B., L.H., J.G.R. conceived and are responsible for creating, developing, and deploying analysis platforms and computational methods for analyzing genomic data.

Research Program Management
Marcus B. Jones, Ph.D., Lyndon J. Mitnaul, Ph.D.

Contribution: All authors contributed to the management and coordination of all research activities, planning and execution. All authors contributed to the review process for the final version of the manuscript.

Supplementary Figures

Figure S1. Causal estimation of TSH on systolic blood pressure. a.) Plot of causal estimate. b.) Funnel plot showing estimates for single variants

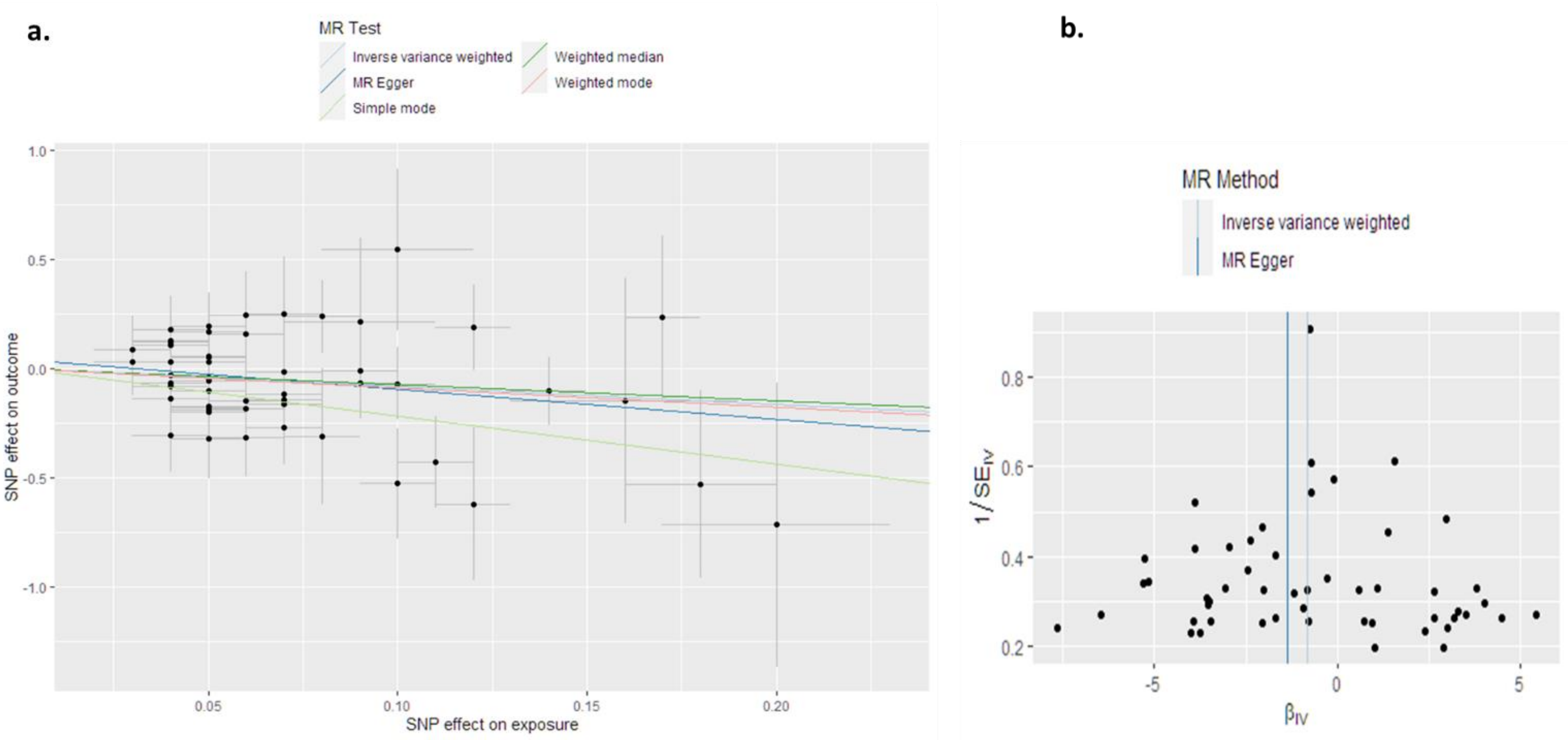

Figure S2 Causal estimation of TSH on atrial fibrillation. a.) Plot of causal estimate. b.) Funnel plot showing estimates for single variants

a.

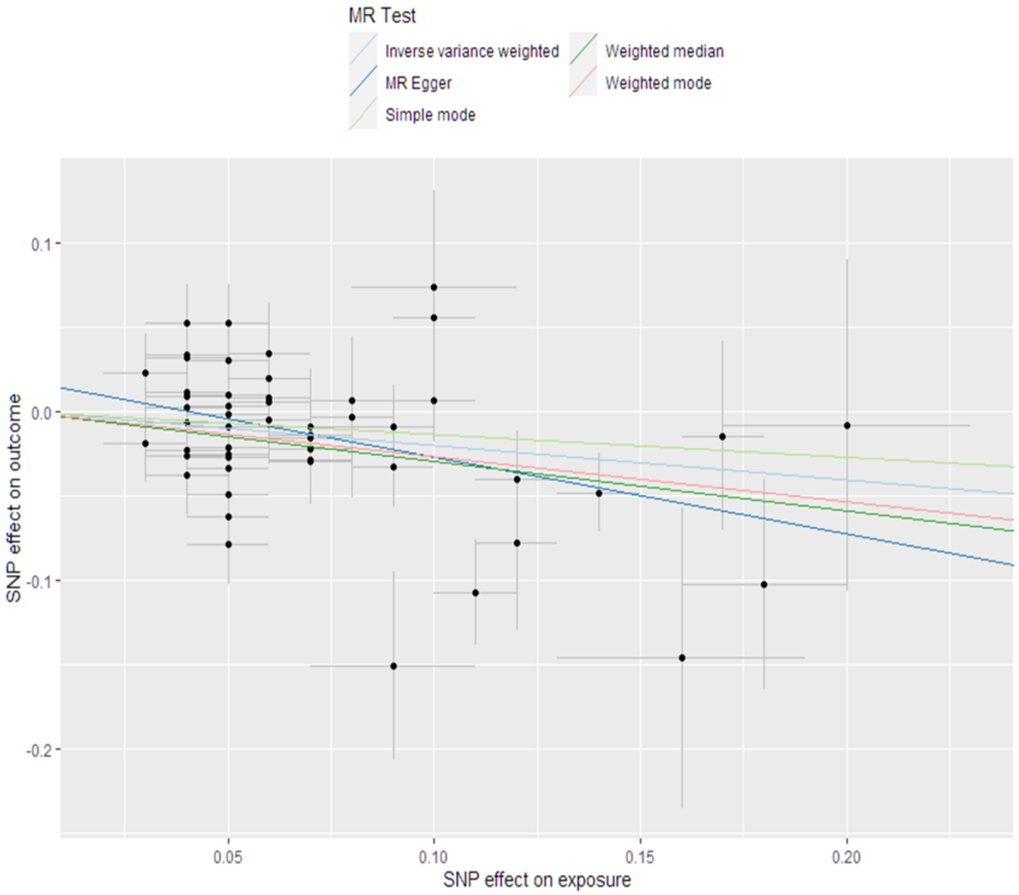

b.

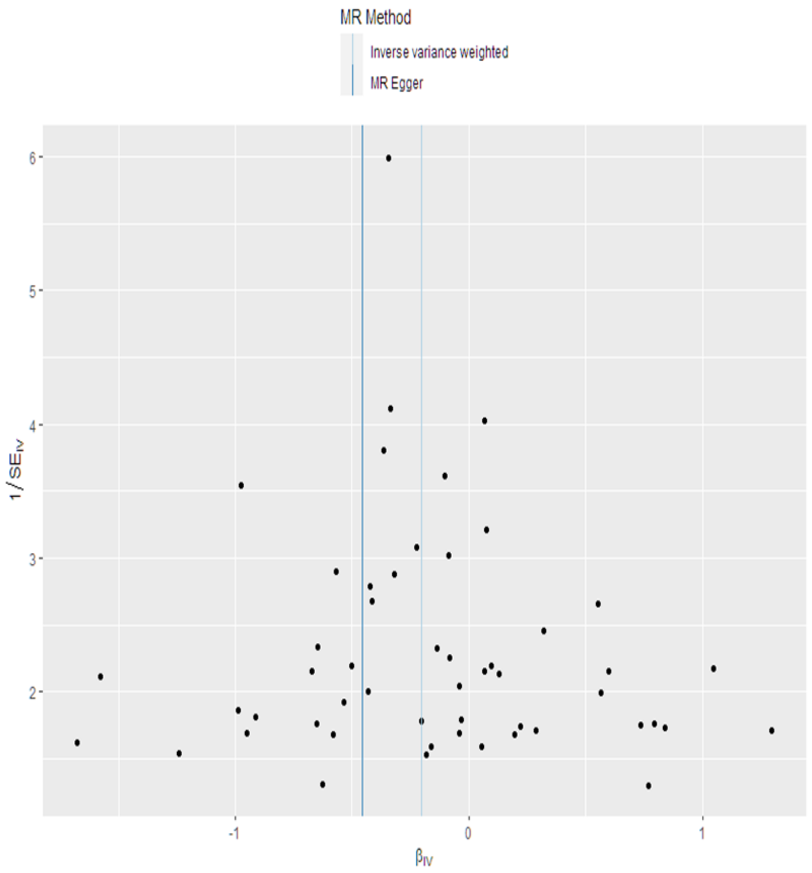

Figure S3 Leave-one-out sensitivity analysis of TSH on SBP

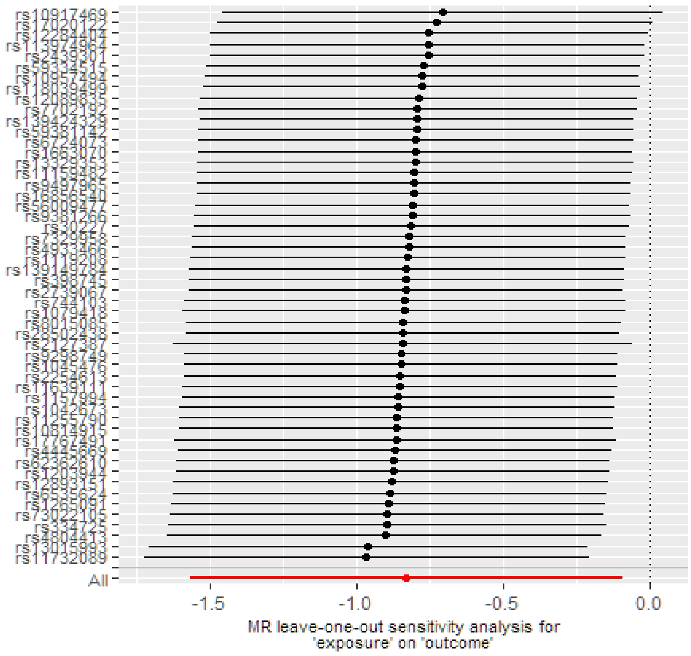

Figure S4 Leave-one-out sensitivity analysis of TSH on AF

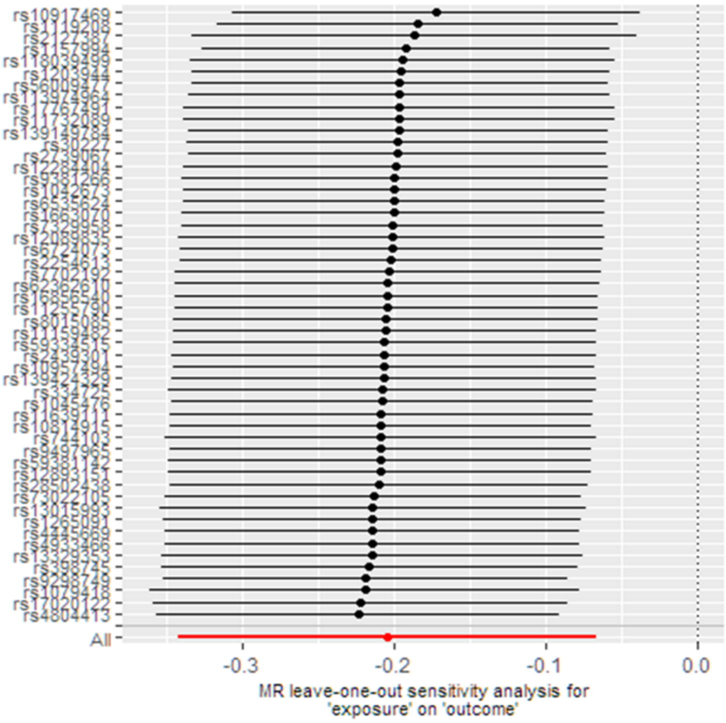

Supplement: Supplementary file 1 [file jpm-11-01306-s001.zip › jpm-1431746_PR_Suppl_File.pdf]
